# Thwarting of Lphn3 Functions in Cell Motility and Signaling by Cancer-Related GAIN Domain Somatic Mutations

**DOI:** 10.3390/cells11121913

**Published:** 2022-06-13

**Authors:** Monserrat Avila-Zozaya, Brenda Rodríguez-Hernández, Feliciano Monterrubio-Ledezma, Bulmaro Cisneros, Antony A. Boucard

**Affiliations:** 1 Department of Cell Biology, Centro de Investigación y de Estudios Avanzados del Instituto Politécnico Nacional (Cinvestav-IPN), Mexico City 07360, Mexico; mavilaz@cinvestav.mx (M.A.-Z.); rodriguez.hernandez@cinvestav.mx (B.R.-H.); 2 Department of Genetics and Molecular Biology, Centro de Investigación y de Estudios Avanzados del Instituto Politécnico Nacional (Cinvestav-IPN), Mexico City 07360, Mexico; eduardo.monterrubio@cinvestav.mx (F.M.-L.); bcisnero@cinvestav.mx (B.C.)

**Keywords:** G protein-coupled receptor, latrophilin, adhesion molecules, cancer somatic mutations, GAIN domain, cell motility, actin cytoskeleton, G protein signaling

## Abstract

Cancer progression relies on cellular transition states accompanied by changes in the functionality of adhesion molecules. The gene for adhesion G protein-coupled receptor latrophilin-3 (aGPCR Lphn3 or ADGRL3) is targeted by tumor-specific somatic mutations predominantly affecting the conserved GAIN domain where most aGPCRs are cleaved. However, it is unclear how these GAIN domain-altering mutations impact Lphn3 function. Here, we studied Lphn3 cancer-related mutations as a proxy for revealing unknown GAIN domain functions. We found that while intra-GAIN cleavage efficiency was unaltered, most mutations produced a ligand-specific impairment of Lphn3 intercellular adhesion profile paralleled by an increase in cell-matrix actin-dependent contact structures for cells expressing the select S810L mutation. Aberrant remodeling of the intermediate filament vimentin, which was found to coincide with Lphn3-induced modification of nuclear morphology, had less impact on the nuclei of S810L expressing cells. Notoriously, receptor signaling through G13 protein was deficient for all variants bearing non-homologous amino acid substitutions, including the S810L variant. Analysis of cell migration paradigms revealed a non-cell-autonomous impairment in collective cell migration indistinctly of Lphn3 or its cancer-related variants expression, while cell-autonomous motility was potentiated in the presence of Lphn3, but this effect was abolished in S810L GAIN mutant-expressing cells. These data identify the GAIN domain as an important regulator of Lphn3-dependent cell motility, thus furthering our understanding of cellular and molecular events linking Lphn3 genetic somatic mutations to cancer-relevant pathogenesis mechanisms.

## 1. Introduction

Adhesion events that dictate the different steps of carcinogenesis are regulated by a complex network of proteins identified as the adhesome. Functional aspects of the adhesome encompass two distinct modes of action classified as mechanical, which involve a direct interaction with the cytoskeleton, or non-mechanical with signaling events linked to cell survival and proliferation. Together, these functions confer cellular plasticity in a process that will improve cancerous transformation through changes in cell shape, migration or invasion [1]. Epithelial-mesenchymal transition (EMT), one of the most studied examples of plasticity as a basis for the oncogenic process, involves the modification of cellular architecture resulting from adhesion molecules dysregulation leading to mismatches in cell-cell or cell-matrix contacts, which will occur throughout cancer development depending on environmental stimuli and cell type [2]. Notably, these constantly changing conditions will instruct which adhesion molecules come into play at a given step of cancer progression.

Latrophilins (Lphns) are a three-member subfamily of widely expressed adhesion G protein-coupled receptors (aGPCRs) participating in various cellular functions ranging from synaptic specificity and embryonic development to inflammation and carcinogenesis [3,4,5,6,7,8,9,10]. As de facto adhesion molecules, Lphns’ heterophilic functions are supported by the presence of extracellular N-terminal adhesion motifs, such as the lectin-like and olfactomedin-like domains, known to stabilize protein-protein interactions with endogenous ligands from the teneurin, neurexin and Fibronectin and Leucine-Rich Transmembrane proteins (Flrt) families [8,11,12,13,14]. A seven-transmembrane spanning segment with interconnecting loops constitute most of the C-terminal region while a signature GPCR autoproteolytic inducing (GAIN) domain separates the N-terminal from the C-terminal. As a self-functioning entity, the GAIN domain comprises two structurally distinct subdomains: subdomain A composed of six α-helices and subdomain B resembling a β-sandwich composed of 13 β-strands and two small α-helices [15]. A cleavage motif known as the GPCR Proteolysis Site (GPS) is embedded into subdomain B before the last β-strand, generating two protomers that remain non-covalently bound to each other during their trafficking from the endoplasmic reticulum to the cell-membrane [15,16]. The last membrane-proximal β-strand acts as a tethered agonist by inserting itself into the GPCR region, whether cleavage at the GPS occurs or not, thus suggesting that the GAIN domain might bear crucial activation determinants [17,18,19,20].

Amidst the different Lphn isoforms, Lphn3 has the most association with human pathologies to date [21]. Stemming from its high expression level in the brain and its role in the formation of synapses, Lphn3 gene defects have been linked to various neuropsychiatric disorders [21,22]. Additionally, its distribution in peripheral tissues allowed for the identification of genetic somatic mutations associated with the presence of cancerous tissue in lung, germinal center B cells and myeloid cells [23,24,25,26]. This oncogenic role was corroborated by the detected overexpression of Lphn3 in cancer cell lines originating from different tissues such as prostate, lung, breast and leukocytes [27]. While it is unknown which signaling pathways would involve Lphn3 functions in cancerous processes, its ability to couple with G13 protein and its actin cytoskeleton remodeling activities are among the functions that are susceptible to being targeted [28,29,30]. Given the potential role of the GAIN domain in aGPCR activation, we sought to investigate the functional defects induced by the presence of single amino acid substitutions into Lphn3 GAIN domain originating from genetic somatic mutations identified in lung cancer primary tumors [25].

## 2. Materials and Methods

### 2.1. Expression Constructs

Lphn3 and Flrt3 expression constructs-plasmids expressing the human Lphn3^HA/Flag^ and the corresponding GAIN domain mutants were previously described [15]. Generation of mVenus-tagged Lphn3 GAIN mutant plasmids was achieved by inserting the EcoRI-BsU36I fragment corresponding to the mutated region for each mutation-containing constructs into the same site of the previously described mVenus-tagged Lphn3^HA/Flag^ [29], thus yielding Lphn3^HA/Flag^-mVenus separately bearing A^760^G (★A760G), K^561^N (★K561N), D^798^H (★D798H), S^810^L (★S810L) or E^811^Q (★E811Q) mutations. Membrane-anchored human Flrt3 construct, as well as soluble Ig-fused human Flrt3 (Flrt3*^ECD^*-Fc) and Teneurin-2 expression plasmids, were previously described [11,31]. TRUPATH BRET-based G13 biosensor was a gift from Bryan Roth (Addgene kit #1000000163).

### 2.2. Cell Culture and Transfections

The HEK293 cell line was obtained from ATCC and routinely tested for presence of mycoplasma. HEK293 cells were cultured in Dulbecco’s Modified Eagle medium (DMEM; Corning) supplemented with 10% fetal bovine serum (FBS; Biowest), 1000 U ml^−1^ penicillin-streptomycin (In Vitro) and 2 mM GlutaMAX (Invitrogen) at 37 °C and 5% CO_2_. Transient transfections were conducted using a 1:3 ratio of transfectant polyethylenimine (PEI, linear, MW 25,000; Polysciences Inc, Warrington, FL, USA) in relation to DNA. Routinely, 1.8 × 10^6^ cells/well of a 6-well plate were treated with a dropwise addition of room temperature DNA-PEI complex (240 µL of DMEM, 4 µg of plasmid DNA and 12 µL of 1 µg/µL PEI incubated for 30 min and then completed to 1 mL with DMEM) and left to incubate at 37 °C for 30 min. Subsequently, 1 mL of DMEM medium supplemented with 20% FBS, 2000 U mL^−1^ penicillin-streptomycin and 4 mM GlutaMAX was added to the cells. Finally, after 19 h of incubation, the media from each well were replaced by 2 mL of DMEM medium supplemented with 10% FBS, 1000 U mL^−1^ penicillin-streptomycin and 2 mM GlutaMAX. For BRET-based biosensor assays cells were co-transfected with 12.5 ng of each plasmid of BRET^2^ biosensor: Gα_13_, Gγ9 and Gβ3; 50, 250 and 750 ng of the Lphn3^HA/Flag^ plasmid and final DNA amount was adjusted to 1000 ng with empty pCMV vector.

### 2.3. Cell Aggregation Assay

HEK293 cells were co-transfected following this scheme: receptor-transfected cells received 0.5 µg of EGFP-encoding plasmid DNA and 3.5 µg of Lphn3 variants plasmid DNA, while ligand-transfected cells received 1 µg of DsRed-encoding plasmid DNA with 3 µg of ligand-encoding plasmids (Teneurin 2 or Flrt3). Forty-eight hours post-transfection, each cell population was harvested using 1 mM ethylene glycol-bis (β-aminoethyl ether)-N,N,N′,N′-tetra acetic acid (EGTA; Sigma-Aldrich, St. Louis, MO, USA) in Phosphate Buffer Saline (PBS; Corning) containing ≥0.01 Kunitz units of DNAse I (Sigma-Aldrich) and thoroughly dissociated through siliconized Pasteur pipettes. Aggregation samples were constituted by mixing receptor-expressing cells with ligand-expressing cells (1:1 ratio in DMEM containing 50 mM HEPES-NaOH pH 7.4, 10% FBS, 10 mM CaCl_2_ and 10 mM MgCl_2_) and incubated for 2 h under constant agitation at room temperature. Aliquots were spotted on microscope slides and images were acquired using a 10× objective of the Observer.Z1 epifluorescence microscope (Zeiss, Göttingen, Germany). Particle analysis conducted with Fiji-ImageJ allowed the determination of their surface area, which were ranked according to single particles found in negative control conditions used as the lower-limit threshold. The aggregation index represented in percentage (%) was obtained by calculating the ratio of the sum areas from particles surpassing the lower-limit threshold over total area occupied by all particles in the field.

### 2.4. Production and Purification of Flrt3^ECD^-Fc

Production of Flrt3*^ECD^*-Fc was performed as reported by [31]. Briefly after 96 h of transfection, HEK293 cell media were collected and cleared of cell debris by centrifugation. To immobilize Flrt3*^ECD^*-Fc, conditioned media were incubated with protein A-coupled sepharose beads (GE healthcare) overnight at 4 °C. After a series of washes, the proteins were eluted from the beads with 4 M MgCl_2_ solution and dialyzed (dialysis buffer: 50 mM Hepes, 150 mM NaCl, pH 7.4) through 100 kDa centrifugal devices (Millipore Sigma, San Diego, CA, USA). The final eluate was analyzed by SDS-PAGE gels and stained with Coomassie blue. Quantification was performed by densitometry analysis using Fiji-ImageJ.

### 2.5. Cell Surface Labeling Assay

HEK293 cells transfected with Lphn3-encoding plasmids were incubated overnight at 4 °C with DMEM containing 10 nM Flrt3*^ECD^*-Fc, 50 mM HEPES-NaOH pH 7.4, 2 mM CaCl_2_ and 2 mM MgCl_2_. Cells were then fixed with 4% paraformaldehyde (PFA; Sigma-Aldrich) for 15 min on ice before conducting a blocking step with PBS containing 3% bovine serum albumin (BSA; Biowest, Riverside, CA, USA) for 1 h at room temperature. Immunodetection of Flag-tagged Lphn3 receptor variants was achieved using polyclonal anti-Flag antibody (1:500 ratio in blocking solution; Sigma-Aldrich, F7425) for 2 h at room temperature and Alexa 633-coupled anti-rabbit secondary antibody (1:500 ratio in blocking solution; ThermoFisher Scientific, Waltham, MA, USA; A21070) for 1 h at room temperature. Bound Flrt3*^ECD^*-Fc was detected using Alexa 488-coupled anti-human Fc antibody (1:500 ratio in blocking solution; ThermoFisher Scientific, Waltham, MA, USA; A11013) for 1 h at room temperature. Nuclei were stained using 300 nM 4′,6-diamidino-2-fenilindol (DAPI; Sigma-Aldrich, 10236276001) in PBS for 5 min. Images were captured by confocal microscopy using the 63× immersion objective (Numerical Aperture [AN]: 1.4) from Leica SP8 microscope (Leica Microsystems, Heidelberg, Germany) and collecting 0.4 µm stacks, which were processed with LAS AF Lite software.

### 2.6. Immunofluorescent Western Blotting

HEK293 cells transfected with plasmids encoding Lphn3 receptor variants were harvested 48 h post-transfection, solubilized in Laemmli sample buffer and resolved by electrophoresis on 10% SDS-PAGE gels before being transferred onto nitrocellulose membranes (Merck-Millipore, Burlington, MA, USA). A blocking step was conducted using 3% BSA in 1× TBST (50 mM Tris pH 7.4, 150 mM NaCl, 0.1% Tween 20) for 2 h at room temperature prior to adding primary antibodies: mouse monoclonal anti-hemagglutinin (HA) (1:1000 ratio in blocking solution; BioLegend, San Diego, MA, USA; 901513), rabbit polyclonal anti-Flag (1:1000 ratio in blocking solution; Sigma-Aldrich, F7425) and mouse monoclonal V9 anti-vimentin antibody (1:250 ratio in blocking solution; Santa Cruz Biotechnology, Dallas, TX, USA; sc-6260) followed by anti-rabbit IRDye680RD and anti-mouse IRDye800CW as secondary antibodies (1:15,000 ratio in blocking solution; LI-COR, 926-68071 and 926-32212, respectively). Finally, membranes were scanned using 700 nm and 800 nm channels from LI-COR Biosciences Odyssey Fc imaging system to quantify fluorescent signals.

### 2.7. Detection of Cell Surface Receptor Expression (DECS Assay)

HEK293 cells transfected with receptor variants-encoding plasmids or with empty vectors were transferred from 6-well plates to poly-L-Lysine (Sigma-Aldrich) treated 96-well plates following 24 h post-transfection and cultured for an additional day. Cells were then fixed with 4% PFA for 10 min on ice and incubated in blocking solution for 1 h at room temperature before adding polyclonal anti-Flag antibody (1:20,000 ratio in blocking solution containing 0.1% sodium azide) followed by an additional hour in the presence of horseradish peroxidase-coupled anti-rabbit secondary antibody (1:2000 ratio in blocking solution; Jackson ImmunoResearch, Baltimore, MD, USA; 111-035-003). The colorimetric reaction was induced by adding horseradish-peroxidase chromogenic substrate, 3,3′,5,5′-tetramethylbenzidine (TMB; Sigma-Aldrich), giving rise to a blue-colored solution that was stopped by adding 1 N H_2_SO_4_ and resulting in a yellow-colored solution. Absorbance at 450 nm was determined using the Cytation5 microplate reader (Biotek, Winooski, VT, USA).

### 2.8. G13 Protein BRET-Based Biosensor Assay

Constitutive Lphn3 activity through the G13 pathway was assessed using the TRUPATH biosensor for G13 (Gα13-RLuc8, Gϒ9-GFP2 and Gβ3) described previously [32]. HEK293 cells (3.5 × 10^5^ cells) were co-transfected with the indicated amounts of individual Lphn3 variants-encoding constructs (0, 50, 250 and 750 ng) alongside the G13 biosensor using a 1:1:1 DNA ratio for each biosensor components (12.5 ng) and distributed equally in 10 wells of a solid white 96-well plate. After 48 h of transfection, cell media were replaced with room temperature BRET buffer (In mM: 10 Hepes pH 7.4, 1 CaCl_2_, 0.5 MgCl_2_, 4.2 KCl, 146 NaCl, 5.5 glucose) and the bioluminescent reaction was initiated by adding 5 µM of the Rluc8 substrate coelenterazine 400a (Goldbio, St. Louis, MO, USA) followed by a 5 min equilibration phase. Emission signals filtered through 515/40 nm and 410/80 nm bandpass filters corresponding to GFP2 and Rluc8 emission wavelength, respectively, were acquired using the microplate reader Cytation 5 (Biotek) and used to determine BRET^2^ ratios as acceptor/donor emission ratios (Em GFP2/Em Rluc8). Thus, in this system, increasing biosensor activation will lead to decreasing BRET^2^ ratios. Given the inversely proportional relationship between the biosensor activation and BRET^2^ ratios, BRET index (iBRET) was arbitrarily implemented to obtain data that reflect direct proportionality between BRET^2^ signals and biosensor activation by reporting the product of the following equation: BRET^2^_(0)_/BRET^2^_(x)_, where BRET^2^ ratio detected at 0 ng of receptor plasmid is divided by BRET^2^ ratio obtained at x ng of receptor plasmid.

### 2.9. Immunocytochemistry

Transfected HEK293 cells were plated onto coverslips treated with conditioned media providing endogenously secreted matrix-attachment factors. Cells were fixed with 4% PFA for 15 min on ice, subsequently permeabilized with 0.1% triton X-100 in PBS for 10 min and incubated in blocking solution for 1 h at room temperature. Vimentin cytoskeleton was immuno-detected with monoclonal antibody V9 recognizing vimentin’s carboxy-terminal residues 411–423 ensuing an incubation of 2 h at room temperature in a humid chamber followed by the addition of Alexa 633-coupled anti-mouse secondary antibody (1:250 ratio in blocking solution; ThermoFisher Scientific, A21052). Filamentous actin (F-actin) staining was concurrently achieved using rhodamine-phalloidin (1:200 ratio in blocking solution containing 1% BSA; Invitrogen, Waltham, MA, USA; A34055) for 1 h at room temperature. Finally, cell nuclei were stained with 300 nM DAPI for 5 min at room temperature. Images were captured using the 100× immersion objective (Numerical Aperture [AN]: 1.3) of Leica SP8 confocal microscope (Leica Microsystems, Heidelberg, Germany) collecting 0.4 µm horizontal stacks and processed with LAS AF Lite software.

### 2.10. Wound-Healing Assay

Prior to transfection, HEK293 cells were seeded in wells pretreated with 0.2% bovine gelatin solution dissolved in PBS (Sigma-Aldrich). After 40 h of transfection, cells were incubated in a starvation medium (DMEM supplemented with 0.5% BSA and 2mM GlutaMAX) for 4 h. Cell proliferation was inhibited by a 2 h incubation with mitomycin [4 µg/mL] before scratching the cell monolayer with a 10 µL universal micropipette tip. Wells were washed once with PBS to remove cell debris and media were replaced with DMEM medium supplemented with 1% FBS, 1000 U mL^−1^ penicillin-streptomycin and 2 mM GlutaMAX. Analysis was performed by collecting images every 12 h through a 10× objective (numerical aperture [AN]: 0.25) of an inverted Leica microscope model DM IL HC LED for transmitted light brightfield and fluorescence (LEICA). Fluorescence and wound area calculations were performed with Fiji-ImageJ.

### 2.11. Live-Cell Imaging of 2D Individual Cell Motility Assays

HEK293 cells (3.6 × 10^5^ cells) expressing the indicated mVenus-tagged receptor variants were transferred from 6-well plates to conditioned media-coated 35 mm glass bottom plates and cultured until they were analyzed by confocal microscopy starting at 45 h post-transfection. Live-cell image acquisition was performed with the Nikon ECLIPSE Ti confocal microscope while preserving initial cell culture conditions and by maintaining the following parameters: fluorescence images were captured using a 20× objective (Numerical Aperture [AN]:0.45) and collecting 3.5 µm horizontal stacks at 10 min intervals for a 3 h time-period or every 5 min for a 1 h time-period (Lphn3 with Flrt3*^ECD^*-Fc) (Excitation 475 nm, emission 509 nm). For assays involving the addition of ligand Flrt3*^ECD^*-Fc [10 nM], image acquisition was initiated 1 h prior adding the ligand and resumed for an additional 2 h. The images were processed with Fiji-ImageJ through the Manual Tracking plug-in using the centering correction tool to monitor cell trajectories. The program calibration parameters were as follows: time interval = 10 min or 5 min (according to the condition), x/y calibration = 0.62 µm. Subsequently, data were analyzed through the chemotaxis and migration tool to obtain values for cell velocity as well as the accumulated and Euclidean distances. Trajectories were adjusted to the intersection point of the x and y axes and plotted on trajectory plots. Accumulated distance represents the total trajectory of the cell, whereas Euclidean distance is defined as the shortest distance between the origin and destination. Cell velocity was defined as the distance traveled for a given time-period.

### 2.12. Image Analysis

For F-actin analysis, maximum projection images were used to measure cell and nuclear dimension parameters, quantification of F-actin structures, and colocalization between F-actin and Lphn3 variants coupled to mVenus. Cell and nuclear dimension data were obtained manually using the Leica software polygonal tool (Leica LAS AF Lite 3.3.10134.0). Filopodia, lamellipodia and blebs were manually identified on the criteria described by [29]. Briefly, Filopodia, rod-shaped protrusions originating from the cell membrane and filled with cortical F-actin; lamellipodia, leaf-shaped protrusions with a base measuring more than 6 µm, and which must contain cortical F-actin at their periphery; blebs, round protrusions with a base measuring less than 2 µm, and which may or may not contain cortical F-actin at their edge. The cell populations analyzed were as follows: Ctrl *n* = 30, ★Lphn3-WT *n* = 38, ★K561N *n* = 39, ★A760G *n* = 33, ★D798H *n* = 34, ★S810L *n* = 31, ★E811Q *n* = 32. Nuclear circularity was calculated as published by [32] as follows: nuclear circularity= 4π (area)/(perimeter) ^2^. Therefore, values close to 1 indicate high circularity.

Vimentin structures were classified and identified manually as reported [33]. Ring-like structures described vimentin filaments surrounding the nucleus and forming nuclear deformations, while knot-like structures were characterized by the absence of filamentous vimentin and adopting a balloon-like shape near the nucleus. The deformation index was obtained by dividing the sum of concave areas within one nucleus over the total nucleus area [34] using the Leica software polygonal tool (Leica LAS AF Lite 3.3.10134.0). Thus, higher ratios represented higher nuclei deformation. The cell populations analyzed were as follows: Ctrl *n* = 29, ★Lphn3-WT *n* = 36, ★K561N *n* = 33, ★A760G *n* = 30, ★D798H *n* = 31, ★S810Lm *n* = 29, ★E811Q *n* = 29.

### 2.13. Statistical Analysis

Data are expressed as means ± standard error of means (S.E.M.) of at least three independent experiments. Statistical analysis was performed with GraphPad Prism version 6.0 using one-way ANOVA, two-way ANOVA or unpaired *t* test followed by a Dunnett’s or Tukey’s test. *p* values are indicated in figure legends.

## 3. Results and Discussion

### 3.1. Cancer-Related GAIN Domain Mutations Alter Heterotypic Lphn3-Mediated Intercellular Adhesion Profile

**Figure 1:**
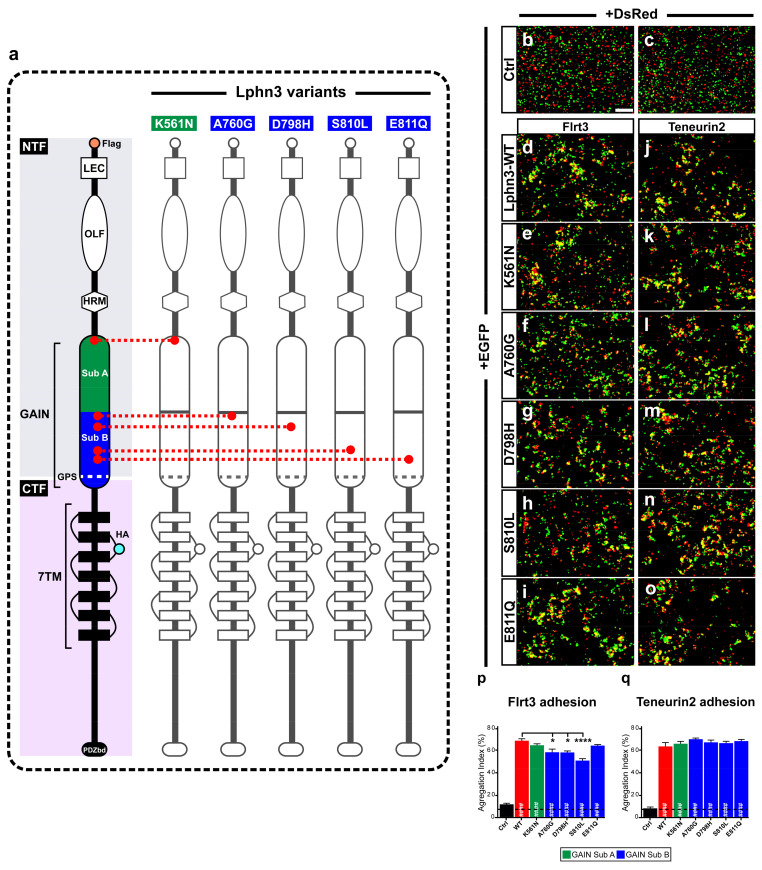
Intercellular adhesion mediated by Lphn3 heterophilic contacts with its ligands Flrt3 and Ten2 is differentially altered by cancer-related GAIN domain mutations. (**a**) Schematic representation of Lphn3 domains organization, depicting cancer-related GAIN domain mutations (K561N, A760G, D798H, S810L and E811Q), Flag tag fused to the amino-terminal, and hemagglutinin tag (HA) introduced in the first extracellular loop. Domain legends: amino-terminal fragment (NTF), carboxyl-terminal fragment (CTF), Lectin (LEC), Olfacftomedin (OLF), hormone binding domain (HRM), GPCR proteolysis site (GPS), seven transmembrane domains (7TM), PDZ-binding domain (P.DZbd). Representative epifluorescence microscopy images of aggregation assays generated by mixing indicated cell populations separately transfected with (**b**,**c**) DsRed or EGFP alone, or (**d**–**o**) in combination with EGFP for Lphn3-WT and indicated Lphn3 variants alongside (**d**–**i**) cells co-expressing DsRed and Flrt3 or (**j**–**o**) Teneurin2. (**p**,**q**) Quantification of aggregation index calculated from assays conducted in between Lphn3-expressing cells and Flrt3- or Teneurin2-expressing cells, respectively. Scale bar: 50 µm. Subdomain A (GAIN Sub A) and B (GAIN Sub B) of GAIN domain. Data are represented as mean values of three independent experiments (*n* = 3). Dotted line represents the values obtained in control conditions (Ctrl). Statistical analysis was performed using one-way ANOVA. Error bars indicate S.E.M., *p* values between Lphn3-variants and control data are indicated by # inside histograms while *p* values between Lphn3-variants and Lphn3-WT are indicated by *: #### or **** *p* ≤ 0.0001, * *p* ≤ 0.05.

**Figure 2:**
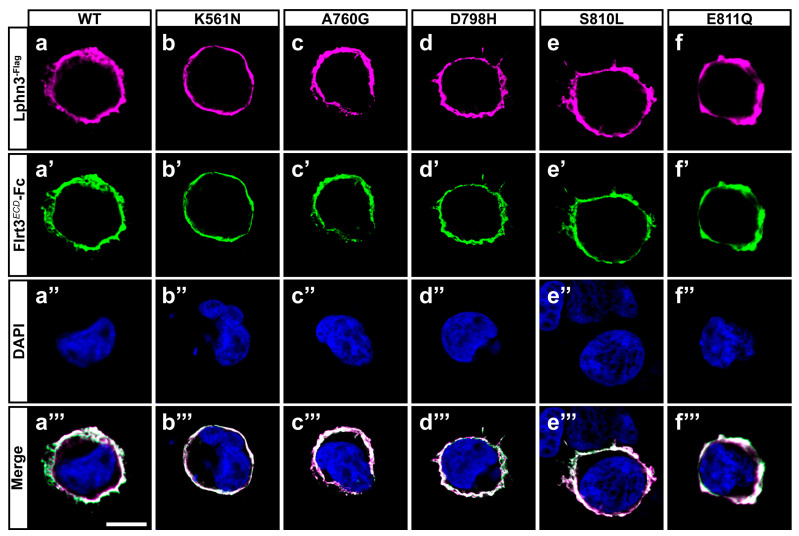
Cancer-related GAIN domain mutations do not abrogate Lphn3 interaction with Flrt3. Representative images of cell surface labeling assays conducted by incubating HEK293 cells expressing Lphn3 or its variants with a soluble recombinant protein corresponding to the extracellular region of Flrt3 fused to IgG constant fraction (Flrt3*^ECD^*-Fc). Cells were visualized by confocal microscopy using (**a**–**f**) anti-Flag antibody (red fluorescent signal from secondary antibody coupled to Alexa 633) recognizing Lphn3 N-terminal, (**a’**–**f’**) anti-human IgG antibody (green fluorescent signal from secondary antibody coupled to Alexa 488) recognizing surface-bound Flrt3*^ECD^*-Fc, (**a’’**–**f’’**) DAPI for nuclei staining. (**a’’’**–**f’’’**) Merged images from corresponding series. Scale bar: 10 µm.

The modulation of intercellular adhesion events plays an important part in defining different phases of carcinogenesis progression by regulating communication between cancer cells and their environment. A change in cancer cells adhesion profile can stem from the pathological modulation of adhesion molecules’ functions either by inducing or downregulating their expression levels or by generating genetic mutational events as previously reported in cancer samples for the aGPCR Lphn3 and its endogenous ligands [25,27]. Given that Lphn3 adhesion functions are mediated through heterophilic ligand-receptor interactions stabilized by its extracellular adhesion domains, we focused on cancer-related mutations specifically located in Lphn3 extracellular GAIN domain (Figure 1a) to investigate their impact on the receptor’s intercellular adhesive properties involving endogenous transmembrane ligands, Flrt3 and Teneurin2. Although Flrt3 and Teneurin2 interact with Lphn3 olfactomedin-like and lectin-like domains, respectively, extracellular mutations have been reported to impair Lphn3-mediated cell-cell adhesion despite being located outside of the receptor-ligand interface [30]. In order to assess the impact of these mutations on Lphn3-mediated heterotypic cell adhesion profile, we monitored the formation of cell-cell contacts through the implementation of cell aggregation assays, using heterologous expression in HEK293 cells, and which consisted of the mixing of Lphn3 variants-expressing cells with Flrt3/Teneurin2-expressing cells. Thus, cells will form aggregates when junctions are stabilized by favorable protein-protein interactions or will be observed as single-cell dispersions when interactions are weak or non-existent (Figure 1b–o). Co-expression of fluorescent proteins differentially identifying each cell population (EGFP for Lphn3 variants-expressing cells and DsRed for ligand-expressing cells) allowed for their visualization with epifluorescence microscopy. In contrast to control conditions conducted with cells lacking the expression of receptor-ligand pairs and consequently displayed a single cell dispersion pattern, Lphn3WT-expressing cells promoted the formation of large cell aggregates when incubated in the presence of either Flrt3- or Ten2-expressing cells. A ligand-specific impact on cell adhesion could be detected for all cancer-related Lphn3 variants, but mainly showed significance for A760G, D798H and S810L as they significantly inhibited the formation of cell-cell junctions with Flrt3-mediated interactions, but not when in contact with Ten2-expressing cells (Figure 1p,q). These results were surprising given that the Lphn3 binding determinants for Flrt3 do not comprise the GAIN domain, but rather the Olfactomedin domain. These unexpected results prompted us to investigate whether the decrease in adhesion was related to a loss of Flrt3 binding to Lphn3 variants. Flrt3-Lphn3 interactions were monitored by conducting cell surface labeling assays, which consisted of the incubation of Lphn3 variants-expressing cells in the presence of soluble Flrt3, devoid of its transmembrane domain and coupled to IgG-Fc, followed by immunodetection of membrane-bound ligand (Figure 2, Supplementary Figure S1). Coincident immunofluorescence receptor labeling corresponding to Flrt3*^ECD^*-Fc was equally detected on plasma membranes of cells expressing wild-type and mutated Lphn3 receptors, thus suggesting that GAIN domain mutations did not abrogate ligand-receptor interactions. Taken together, these results point to a description of Lphn3-mediated intercellular adhesion that does not strictly depend on receptor-ligand interactions, but might involve additional factors, which preferentially destabilize Flrt3- and not Teneurin2-dependent adhesion complexes. Such factors are likely to originate from the receptors’ intrinsic conformational states thought to allow the conversion of mechanical/adhesive stimuli into the activation of intracellular signaling pathways, a feature attributed to its CTF rather than its NTF, and previously described to explain constitutive, as well as ligand-dependent activity of Lphn3 [30,35,36]. The ability of Lphn3 to signal through both G protein-dependent and -independent pathways towards the actin cytoskeleton raises the possibility that its adhesive units might rely on cytoskeletal anchoring to sustain tensile forces, such as the ones detected in aggregation assays, during which shear forces are generated. Thus, NTF-mediated interactions with soluble ligands would remain unchanged in contrast to transmembrane ligands that are more apt at generating transcellular forces. Consequently, faulty transcellular adhesive units with Flrt3 observed for GAIN domain receptor variants could be due to insufficient cytoskeletal anchoring points when elicited through the olfactomedin domain. This would suggest that the GAIN domain has a modulatory function in the ligand-dependent adhesion profile of Lphn3 through signaling mechanisms.

### 3.2. Unaltered Lphn3 Autoproteolysis Efficiency and Expression Amidst the Presence of GAIN Domain-Modifying Mutations

**Figure 3:**
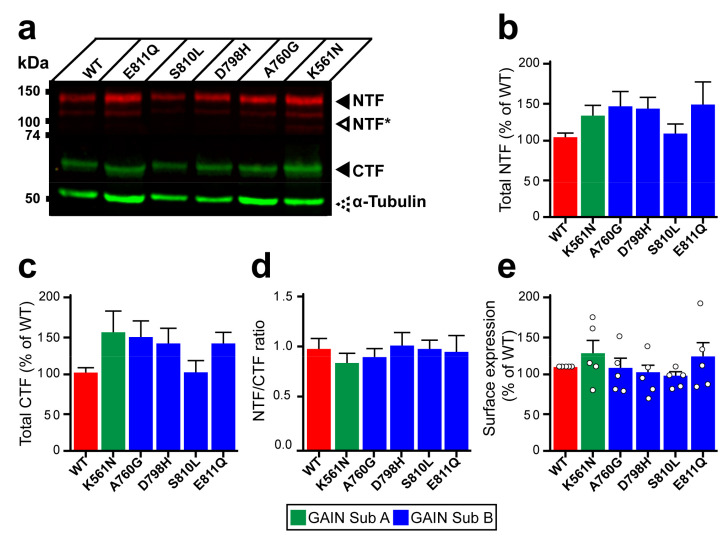
Expression and autoproteolysis of Lphn3 are unaltered by the presence of cancer-related GAIN domain mutations. (**a**) Western blot analysis by immunofluorescent detection of total proteins from HEK293 cells transfected with cancer-related GAIN domain mutations. NTF was detected with a rabbit anti-Flag antibody and CTF with a mouse anti-HA antibody, both distinctly labeled with corresponding secondary fluorescent antibodies. NTF* represent fragments resulting from unknown posttranslational modifications. Immunodetection of α-tubulin was used as a protein loading control. (**b**,**c**) Quantification of normalized fluorescent immunodetection data obtained in (**a**) for NTF and CTF, respectively. (**d**) Cleavage efficiency quantification expressed as NTF/CTF ratios. Data from b-d are represented as the mean values obtained from at least four independent experiments with error bars representing S.E.M. (*n* = 4). (**e**) Detection of cell surface expression assay (DECS) for Flag-tagged Lphn3 and cancer-related GAIN domain mutations using a colorimetric reaction quantifying the acid-stopped conversion of Horseradish-peroxidase substrate, which is detected at a 450 nm absorbance wavelength. Subdomain A (GAIN Sub A) and B (GAIN Sub B) of GAIN domain. Statistical analysis was performed using one-way ANOVA. Each white circle symbol represents the mean value of four replicates obtained from one independent experiment. Data were normalized to Lphn3-WT and are represented as the mean values obtained from at least five independent experiments of four replicates, each with error bars representing S.E.M. (*n* = 5).

Structural organization of the GAIN domain into two subdomains prompted us to investigate whether mutations present in subdomain A (K561N) versus subdomain B (A760G, D798H, S810L and E811Q) had differential effects on autoproteolysis at the GPS site located within the latter (Figure 1a). Whole cell lysates from HEK293 cells expressing Lphn3-WT or its cancer-related variants were analyzed by immunoblotting in order to detect the N-terminal fragment (NTF) through the Flag epitope fused to the Lectin domain and the C-terminal fragment (CTF) through the Hemagglutinin tag inserted in extracellular loop 1 (Figure 3a, Supplementary Figure S2a). Quantification of fluorescence-coupled immunodetection for NTF and CTF levels revealed similar values for all receptor variants compared to Lphn3-WT, thus indicating that the production of GPS-generated fragments was unaltered by GAIN domain mutations (Figure 3b,c). Cleavage efficiency, evaluated through NTF/CTF ratios, was found to be unaffected for receptor variants when compared to Lphn3-WT (Figure 3d). While these data suggested that receptor biogenesis reached wild-type levels for all Lphn3 variants, an assessment of their specific insertion into the plasma membrane was conducted in order to monitor their correct folding, which would result in their proper trafficking to this subcellular compartment where GPCRs exert most of their functions. Immunodetection of the extracellularly exposed Flag epitope from non-permeabilized transfected cells yielded comparable signals between all receptor variants and Lphn3-WT expressing cells, thus indicating similar cell surface expression levels (Figure 3e). Therefore, the propensity of Lphn3 to form adhesion complexes is maintained despite the presence of GAIN domain mutations and does not contribute to the attenuation of Flrt3-mediated intercellular adhesion observed for select cancer-related variants.

### 3.3. Intrinsic Lphn3 Signaling through G13 Protein Activation Is Hindered by Select Cancer-Related Mutations Affecting Both GAIN Subdomain A and Subdomain B

**Figure 4:**
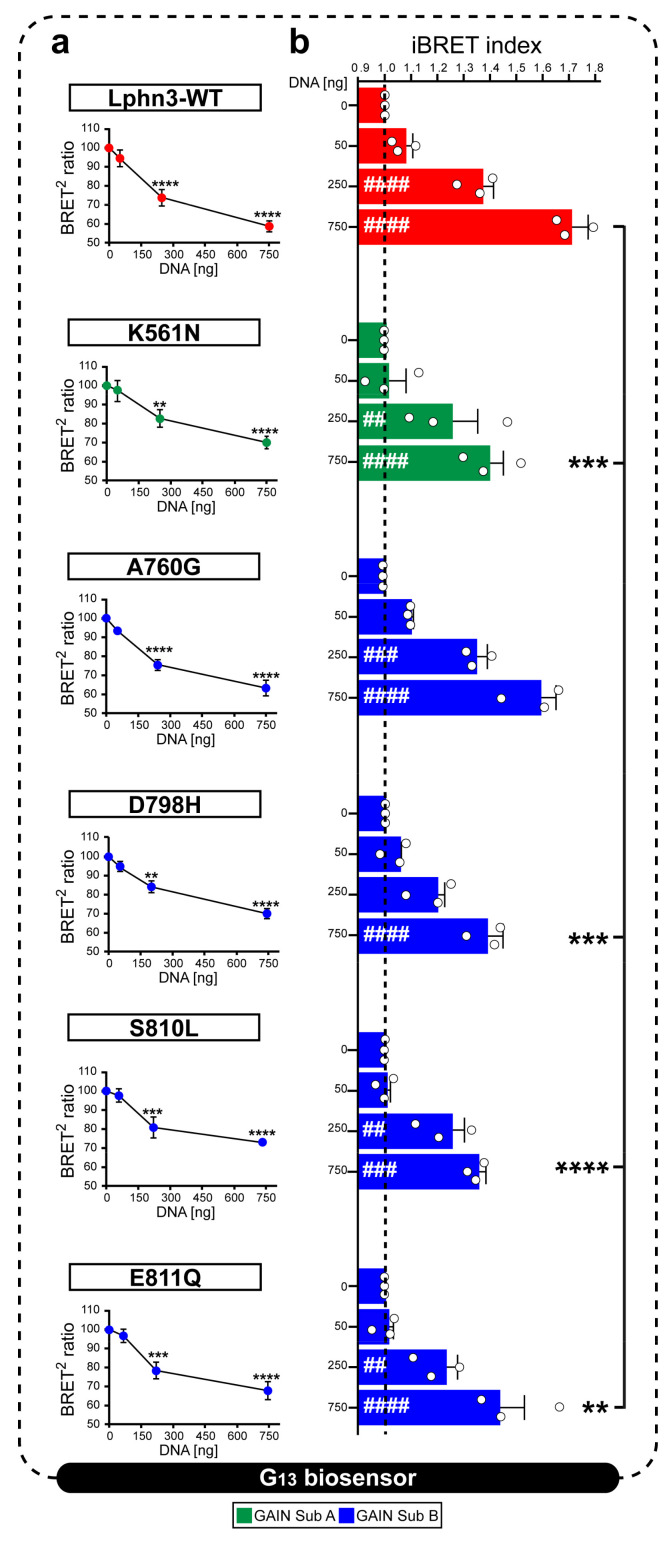
Lphn3 signaling through G13 protein is impaired by cancer-related GAIN domain mutations K561N, D798H, S810L and E811Q but not A760G. Constitutive receptor activity was modulated by co-transfecting HEK293 cells with increasing amounts of Lphn3 receptor plasmids (0, 50, 250, 750 ng) while maintaining a constant concentration of Gα13 biosensor constructs (Rluc8-Gα13, Gβ3, GFP2-Gϒ9). (**a**) Normalized plots reporting receptor DNA concentration-dependent activation of BRET-based Gα13 biosensor. Decreasing BRET^2^ ratio values indicates an increase in the activity of Gα13 biosensor. Normalization was achieved by comparing given BRET^2^ ratios to the maximum BRET^2^ (set at 100%) obtained in conditions where only the biosensor was expressed. *p* values describing the significance between normalized BRET^2^ ratio values obtained at zero ng and a given plasmid concentration of the same receptor: **** *p* ≤ 0.0001, *** *p* ≤ 0.001, ** *p* ≤ 0.01. (**b**) iBRET index obtained for indicated receptors DNA amounts, for which an increase in iBRET corresponds to the increased activity of the biosensor. Subdomain A (GAIN Sub A) and B (GAIN Sub B) of GAIN domain. Each white circle symbol represents the mean value of three replicates obtained from one individual experiment. Data are represented as the mean values of at least three independent experiments of four replicates each (*n* = 3). Statistical analysis was performed using two-way ANOVA. Error bars indicate S.E.M., *p* values between 0 ng DNA (black dotted line) and each DNA concentration of the same Lphn3-variant are indicated by # inside histograms, while *p* between 750 ng DNA of Lphn3-variants and 750 ng DNA of Lphn3-WT are indicated by *: #### or **** *p* ≤ 0.0001, ### or *** *p* ≤ 0.001, ## or ** *p* ≤ 0.01.

An inherent property of the Lphn3 receptor resides in its ability to activate cell-autonomous intracellular signaling cascades in a ligand-independent manner notably by coupling to various families of G proteins, but preferably to the G13 family subset [30]. Lphn3 GAIN domain is known to encrypt a tethered ligand that can interact with the CTF’s transmembrane segments through various degrees of exposure made possible by dynamic rearrangements of the GAIN domain itself [37]. Despite the tethered ligand hypothesis still representing a contentious matter, we were prompted to verify if mutations to the GAIN domain harbored by cancer-related variants could inherently destabilize its activity-inducing conformation toward G13 protein coupling. Activation of the G13 pathway was monitored using a biosensor based in bioluminescence resonance energy transfer (BRET) accounting for the proximity-dependent fluorescent signal initiated by light emission from luciferase Rluc8 fused to the Gα13 subunit exciting green fluorescent protein GFP2 fused to Gϒ9 subunit [38]. In an attempt to assess receptor constitutive activity, HEK293 cells were transfected with increasing amounts of plasmid DNA encoding Lphn3 variants to gradually promote the formation of receptor-G protein complexes, while maintaining biosensor DNA constant throughout (Figure 4a). The decrease in normalized BRET^2^ signals and their conversion into increasing iBRET signals indicated that Lphn3-WT expressing cells displayed an efficient activation of the G13 biosensor, which was dependent on plasmid DNA amount, a feature of constitutive activity (Figure 4a,b). Every cancer-related receptor variant exhibited constitutive activity, however K561N, D798H, S810L and E811Q mutations significantly decreased Lphn3 maximal efficiency at coupling to the G13 pathway. Notably, the only mutation resulting from a homologous amino acid substitution, A760G, did not significantly affect Lphn3 signaling through G13. Given that actin cytoskeleton remodeling can be modulated by the activation of the G13 pathway, the functional deficiency observed for receptor variants suggests that alterations of both GAIN subdomain A and B caused by somatic mutations within the Lphn3 gene can contribute to cell signaling impairments linked to actin dynamic defects during carcinogenesis.

### 3.4. Lphn3-Induced Inhibition of Lamellipodia Formation Is Prevented in Cells Expressing S810L Cancer-Related Variant

**Figure 5:**
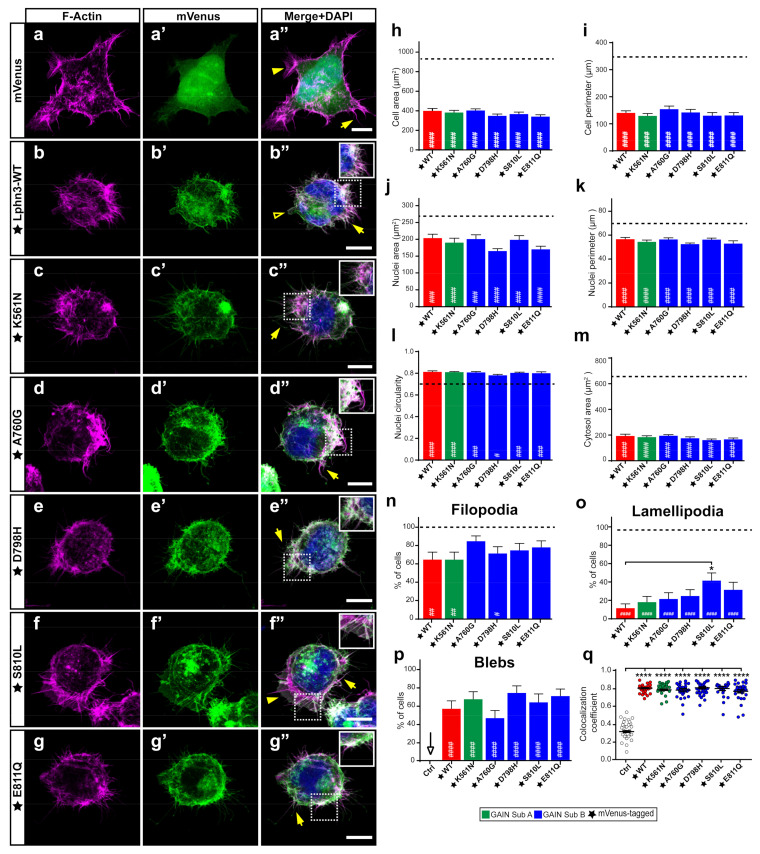
Lphn3-mediated actin remodeling is altered by the presence of S810L mutation. Representative confocal fluorescence microscopy images of HEK293 cells expressing (**a’**–**g’**) mVenus (Ctrl) or the indicated mVenus-tagged Lphn3 variants (green), for which (**a**–**g**) F-actin was stained with phalloidin rhodamine (magenta) and (**a’’**–**g’’**) DAPI for nuclei (blue) in corresponding merged images. Inset images show colocalization of F-actin with the receptor represented as white pixels. Actin-dependent extensions are indicated as follows: filopodia (arrow), lamellipodia (arrowhead) and blebs (empty arrowhead). Scale bar: 10 µm. (**h**–**m**) Quantification of the different cellular and nuclear dimensions. (**n**–**p**) Quantitation of actin-dependent extensions present in transfected cells. Dotted line represents values obtained for mVenus-expressing cells used as control. (**q**) Pearson’s coefficient scatter plot representing coincident pixels between F-actin rhodamine signal and fluorescence emitted by mVenus-tagged Lphn3 variants. Subdomain A (GAIN Sub A) and B (GAIN Sub B) of GAIN domain. mVenus-tagged receptor constructs are indicated by a star (★). Data are represented as the mean values of at least three independent experiments (*n* = 3). At least 30 cells were analyzed for each condition. Statistical analysis was performed using one-way ANOVA. Error bars indicate S.E.M., *p* values between mVenus-tagged Lphn3-variants and control data are indicated by # inside histograms, while *p* values between mVenus-tagged Lphn3-variants and Lphn3-WT are indicated by *: #### or **** *p* ≤ 0.0001, ### *p* ≤ 0.001, ## *p* ≤ 0.01, # or * *p* ≤ 0.05.

The stabilization of intercellular adhesion junctions relies on components of the cell cytoskeleton acting in conjunction with adhesion molecules to assemble intracellular signaling complexes. We have previously evidenced that the actin cytoskeleton of cells expressing Lphn3 suffered changes affecting cell and nuclei morphologies as well as the formation of actin-dependent migratory protrusions pointing to an adherence deficiency phenotype [29]. Both G protein-dependent and G protein-independent mechanisms accounted for Lphn3 functional interaction with the actin cytoskeleton, with the NTF mainly affecting cell and nuclei size parameters. Thus, we were interested in investigating the impact of NTF-located GAIN domain variants on cell and nuclei morphology as well as on the presence of actin-dependent structures related to cell migration. Therefore, cells expressing mVenus-tagged versions of Lphn3 variants were stained with DAPI to identify nuclei and rhodamine-labeled phalloidin to visualize their filamentous-actin (F-actin) content and allow delimitation of the cell plasma membrane through detection of cortical actin (Figure 5a–g). Confocal microscopy image analysis confirmed the coincident detection of Lphn3-WT with the cortical actin (Figure 5a’’–g’’,q) supporting membrane localization along with decreased cell/nuclear area and perimeter dimensions, increased nuclei circularity, inhibition of lamellipodia formation and induction of blebs, which were non-existent for control-transfected cells (Figure 5h–p). Lphn3 variants replicated the same cell modifications induced by the wild-type receptor except for S810L-expressing cells, which harbored a significantly higher rate of lamellipodia formation. As lamellipodia formation relies on a positive feedback between integrin-based adhesions and Rac1 activity, as well as a GPCR-independent interplay between Rac1 and G13 [39,40], these results suggest that G13-independent mechanisms are also at play considering that only the S810L exhibited this phenotype among all other G13 signaling-deficient receptor variants.

### 3.5. The Vimentin Cytoskeleton of Lphn3-Expressing Cells Experiences Drastic Alterations Promoting Nuclear Deformations Attenuated by Cancer-Related Receptor Mutations S810L and E811Q

**Figure 6:**
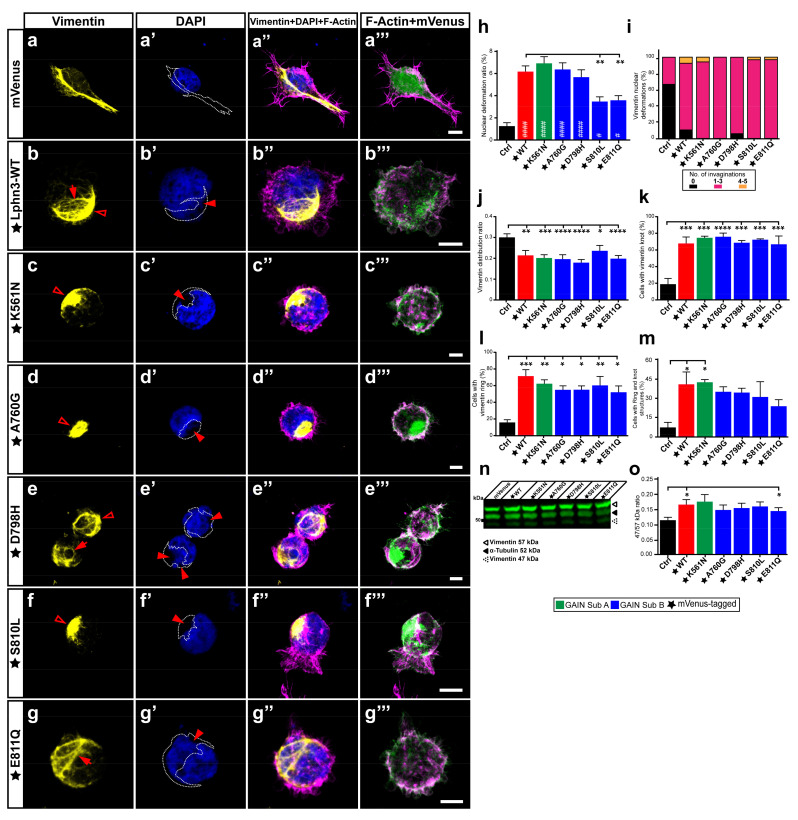
Nuclear deformations coincide with Lphn3-induced vimentin cytoskeleton juxtanuclear packing but are inhibited with S810L and E811Q mutations. Representative confocal microscopy images of HEK293 cells expressing mVenus (Ctrl) or the different mVenus-tagged Lphn3 variants, for which (**a**–**g**) the vimentin cytoskeleton was detected by immunofluorescence with a monoclonal antibody raised against its carboxyl terminus (yellow) and stained for (**a’**–**g’**) nuclei with DAPI (cyan) along with (**a”**–**g”**) F-actin (magenta). In a-g the vimentin ring-like structures are represented with arrows, while empty arrowheads represent the knot-like structures. In (**a’**–**g’**) arrowheads represent nuclear deformation sites and the superimposed coincident vimentin cytoskeleton is delineated by a dotted line. (**a”’**–**g”’**) Merged images of mVenus and F-actin signals. Scale bar: 10 µm. At least 30 cells were analyzed for each condition. (**h**) Analysis of nuclear deformation ratio calculated as nuclear deformation area/nuclear total area. (**i**) Percentage distribution displaying the number of nuclear deformations that coincided with vimentin cytoskeleton. (**j**) Area distribution of vimentin (vimentin area/total cell area). (**k**–**m**) Quantification represented in percentage of cells that presented either one or both knot-like and ring-like structures of the vimentin cytoskeleton. (**n**) Western Blot of total lysates from cells transfected with Lphn3 constructs revealing the immunodetection of vimentin. Note the presence of bands corresponding to full-length vimentin (57 kDa) and cleaved vimentin (47 kDa). Detection of α-tubulin was used as a protein loading control. (**o**) Data of normalized fluorescence ratio between cleaved vimentin over full-length vimentin signal (47/57 kDa). Subdomain A (GAIN Sub A) and B (GAIN Sub B) of GAIN domain. mVenus-tagged receptor constructs are indicated by a star (★). Data are represented as the mean values of at least three independent experiments (*n* = 3). Statistical analysis was performed using one-way ANOVA and *t*-test. Error bars indicate S.E.M., *p* values between mVenus-tagged Lphn3 variants and mVenus control data are indicated by # inside histograms, *p* values between mVenus-tagged Lphn3 variants and Lphn3-WT are indicated by *: #### or **** *p* ≤ 0.0001, *** *p* ≤ 0.001, ** *p* ≤ 0.01, # or * *p* ≤ 0.05.

Adhesion complexes involved in lamellipodia formation often solicit the participation of intermediate filaments in addition to actin filaments. The epithelial-mesenchymal transition, underlining a benign tumor’s ability to acquire infiltrating and metastasizing properties, is marked by an upregulation in cellular levels of the intermediate filament vimentin, which will protect the cancer cells from mechanical stresses during migration by providing an increase in the viscoelastic framework and supporting the integrity of organelles such as the nucleus [41]. This protection mechanism has been visualized by the immunocytochemical description of the vimentin cytoskeleton forming an encaging structure surrounding the cell nucleus resulting in containment-induced pressure points and deformation of the nucleus morphology [42]. Notably, our morphological analysis of multiple nuclei from cells expressing Lphn3 consistently revealed the presence of nuclei malformations with visible lobulations interspersed by invaginations, the nature of which was not immediately clear (Figure 6a–g, Supplementary Figure S3). While comparing Lphn3-expressing cells with control cells, these nuclei deformations were up to five times more prevalent in the former than in the latter (Figure 6h). Further analyses revealed that expression of Lphn3 cancer-related neighboring GAIN mutations S810L and E811Q significantly decreased the presence of these nuclei deformations when compared to Lphn3-WT expression (Figure 6h). Given the intimate contact between vimentin and the nucleus amidst the report that it might represent a marker for epithelial-mesenchymal transition during cancer [43], we sought to visualize the distribution of endogenous vimentin in Lphn3-expressing cells through immunofluorescence confocal microscopy analysis. Control cells expressing mVenus alone displayed an extended vimentin cytoskeleton organized in an extensive filamentous network spanning the perinuclear region and the cytoplasmic area (Figure 6a). This distribution was in stark contrast with the one exhibited by cells expressing Lphn3, in which vimentin formed entangled knot-like structures adjacent to the nucleus and/or formed rings that surrounded nuclear lobulations (Figure 6b, Supplementary Figure S3). Lphn3-WT impact on vimentin cytoskeleton was matched to a similar extent in cells expressing receptor variants (Figure 6c–g). Compaction of the vimentin cytoskeleton was further evidenced by comparing the area it occupied within the cell to total cell area, revealing lower spreading ratios for Lphn3-expressing cells than control cells and thus indicating that the mere reduction in cell area resulting from receptor expression could not by itself explain the alteration in vimentin’s redistribution (Figure 6j). Interestingly, a compact and perinuclear arrangement of the vimentin cytoskeleton was also described in cancer cells with amoeboid-like migration, which was important for moderating the velocity of the leading bleb [44]. Given the protective role of vimentin cytoskeleton toward the nucleus we further analyzed the relationship between both. When the immunofluorescent signal for vimentin was superimposed with the DAPI/nucleus signal, we observed that areas of indentation resulting in a concave nuclear morphology significantly coincided with the presence of entangled vimentin in Lphn3-expressing cells in comparison to control cells, which presented very low occurrence of this phenotype (Figure 6a’–g’,i). While the presence of these compact vimentin structures were 3–4 times more prevalent in Lphn3-expressing cells than in control cells, sometimes even displaying significant overlap with cells exhibiting both structures, we could not detect differences in prevalence of this vimentin organization between Lphn3-WT and mutants (Figure 6k–m). These visual observations of vimentin entanglements in Lphn3-expressing cells clearly suggested a reorganization of this intermediate filament protein into non-filamentous entities, a process that can be driven by proteolytic cleavage of vimentin, and which is associated with the modulation of cell invasion [44]. Immunodetection of endogenous vimentin from total cell extracts of transfected cells revealed the presence of intact vimentin as well as its cleaved product migrating as a 47 kDa band reminiscent of protease-generated vimentin fragments (Supplementary Figure S2b) [45]. Samples obtained from control-transfected cells presented a prominence of uncleaved vimentin, while Lphn3-expressing cells produced a higher cleavage efficiency evidenced by a higher ratio between cleaved and uncleaved protein species with no significant differences in comparison to receptor mutants (Figure 6n). Given the important role played by vimentin cytoskeleton in cell migration, these results would suggest *a priori* that Lphn3 expression or that of its cancer-related variants might be inducing cell migration patterns that significantly diverge from naive cells given their involvement in vimentin cytoskeleton restructuration, disassembly, and spatial redistribution.

### 3.6. Wound-Induced Cell Population-Wide Migration Is Halted as a Result of Lphn3 and Cancer-Related Mutants Receptor Expression

**Figure 7:**
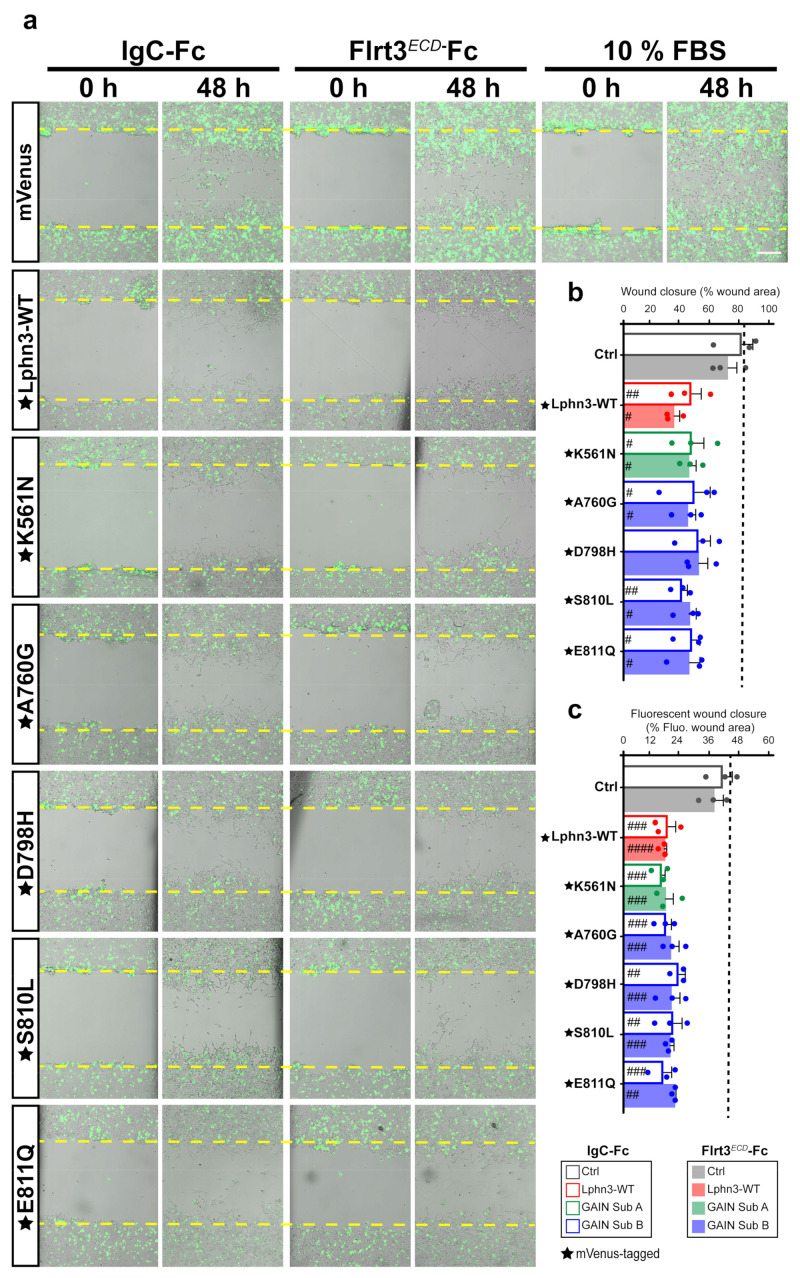
Lphn3-induced delay in cell-autonomous and non-cell-autonomous polarized migration is unperturbed by cancer-related GAIN domain mutations in wound healing assays. (**a**) Representative merged images from bright field and epifluorescence microscopy of mVenus-tagged Lphn3-expressing cells or mVenus control cells depicting wound closure assays captured at the initial scratch into the cell monolayer (zero hours) and at the same scratch area 48 h later, in the absence (IgC-Fc) and presence of receptor ligand Flrt3*^ECD^*-Fc. The yellow dotted lines represent the edges of the scratch at time zero hours. Cells incubated with 10% FBS were used as positive migration controls. Scale bar: 200 µm. (**b**) Percentage scratch closure data in the presence of IgC-Fc or Flrt3*^ECD^*-Fc represented as the percentage of the total scratch area at zero hours that is occupied by cells at 48h, in bright field images. (**c**) Ratio between fluorescence area occupied by cells expressing mVenus signal in the wound (mVenus-tagged receptors or control cells) and total fluorescence area in the field of view. The dotted line represents the data corresponding to positive control migration values. Subdomain A (GAIN Sub A) and B (GAIN Sub B) of GAIN domain. mVenus-tagged receptor constructs are indicated by a star (★). Data are represented as the mean values of at least three independent experiments (*n* = 3). Statistical analysis was performed using one-way ANOVA. Error bars indicate S.E.M., *p* values between mVenus-tagged Lphn3 variants and control data are indicated by # inside histograms: #### *p* ≤ 0.0001, ### *p* ≤ 0.001, ## *p* ≤ 0.01, # *p* ≤ 0.05.

Cell shape dynamics are tightly related to cell migration patterns, which underline events leading to metastasis [46]. Since cells expressing Lphn3 and cancer-related GAIN mutants exhibited an adhesion-deficient phenotype, which contrasted with that of control cells, we tested whether these morphological changes would have an impact on cell migration. Typically, the generation of a wound into a cell monolayer will drive the release of proinflammatory factors and growth factors for wound repair. We sought to implement a wound healing assay in order to analyze the effect of Lphn3 expression on the collective migration of a heterogeneous cell population (Lphn3-expressing and non-expressing cells) in response to repair factors. Transfected cells were treated with mitomycin-C to prevent cell proliferation and the cell monolayer was scratched with a single stroke before being monitored by brightfield and fluorescence microscopy immediately following the insult and up to 48 h later. Brightfield analysis revealed that a nearly complete wound closure was achieved (~80%) for samples corresponding to the positive control containing mVenus-transfected cells and engineered to convey optimal migration conditions by the addition of fetal bovine serum (Figure 7a,b). Incubation of control cells with either Ig-Fc or Flrt3*^ECD^*-Fc produced a similar migration pattern by displaying wound closures of 68 and 69%, respectively, (Figure 7b). Strikingly, Lphn3-transfected cells whether expressing Lphn3 or not, collectively failed to yield control-level wound closure irrespective of their incubation in absence or presence of Flrt3 ligand (33% and 40% respectively), thus suggesting a non-cell-autonomous effect originating from the receptor’s constitutive activity and independent of its ligand-mediated activation (Figure 7b). Samples containing cells transfected with GAIN mutant constructs replicated the wound closure pattern displayed by Lphn3-WT transfected cells, revealing comparable yields. Notably, the monitoring of fluorescent signals to locate Lphn3-expressing cells, unveiled that these cells remained at the border of the wound contrary to control mVenus-expressing cells, which redistributed in an almost aleatory fashion between the border and inner space of the wound (Figure 7c, Supplementary Figure S4). These results suggest that Lphn3 and its variants inherently exert both a cell-autonomous and a non-cell-autonomous regulation on collective cell migration, potentially by desensitizing cells to yet unknown wound-healing factors.

### 3.7. Cell Motility-Inducing Activity of Lphn3 Is Disrupted by GAIN Domain Mutation S810L

**Figure 8:**
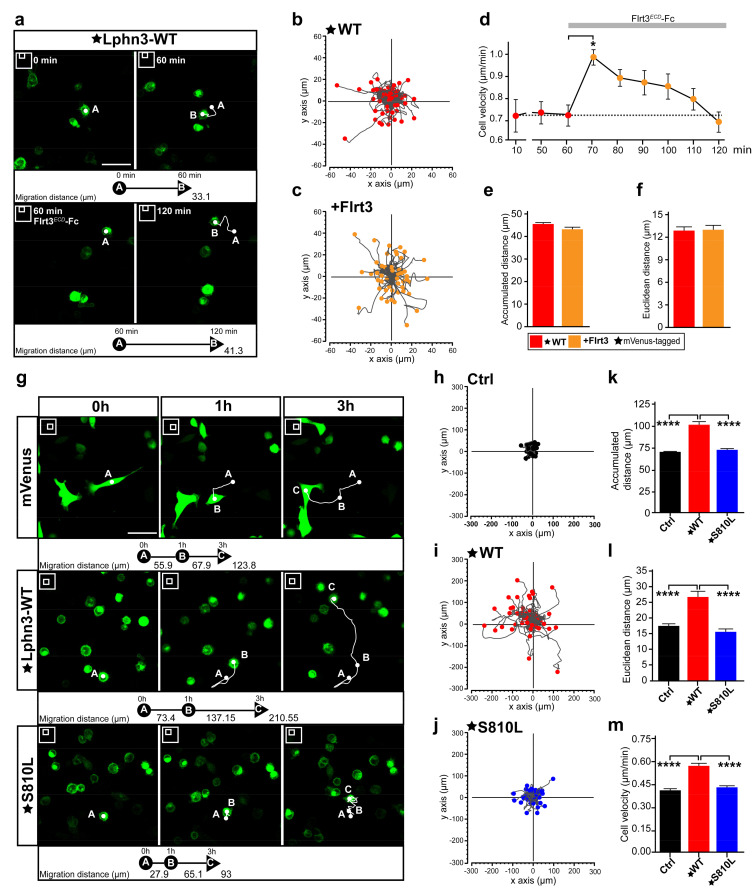
Cell motility is induced by cell-autonomous Lphn3 expression but inhibited by cancer-related GAIN domain mutation S810L. (**a**) Representative images obtained at 5 min interval by time-lapse microscopy of low-confluency HEK293 cell cultures transfected with plasmid encoding mVenus-tagged Lphn3, before and after incubation with Flrt3*^ECD^*-Fc. 2D cell positioning is indicated by A and B at indicated image acquisition time points. White lines represent the cell trajectory. Scale bar: 50 µm. (**b**,**c**) Trajectory plots showing the path of individual cells over 1 h for cells analyzed in A. Tracks were fixed at a common origin (x-y axes intersection) and ended at positions indicated by respective filled circles. (**d**) Time-lapse velocity analysis of mVenus-tagged Lphn3-WT expressing cells stimulated with Flrt3*^ECD^*-Fc. The dotted line represents the baseline of unstimulated cells’ mean velocity. (**e**,**f**) Quantification of 1 h-long monitoring of accumulated and Euclidean distances before (red) and after (orange) incubation with Flrt3*^ECD^*-Fc. Data are represented as the mean values of at least three independent experiments (*n* = 3). A total of 120 cells were analyzed for each condition. Error bars indicate the S.E.M., *p* values in (d) are indicated by *:* *p* ≤ 0.05. (**g**) Representative images obtained at 10 min interval by time-lapse microscopy of low-confluency HEK293 cell cultures transfected with plasmid encoding indicated proteins at the following time points: 0 h, 1 h and 3 h. 2D cell positioning is indicated by A, B and C at indicated image acquisition time points. White lines represent the cell trajectory. Scale bar: 50 µm. (**h**–**j**) Trajectory plots showing the path of individual cells over 3 h with tracks adjusted similar to (B). Between 45 and 55 cells from a randomly chosen field of view were analyzed. (**k**–**m**) Quantification of 3 h-long monitoring of cell velocity, accumulated and Euclidean distances. Subdomain A (GAIN Sub A) and B (GAIN Sub B) of GAIN domain. mVenus-tagged receptor constructs are indicated by a star (★). Data are represented as the mean values of at least three independent experiments (*n* = 3). A total of 159 cells were analyzed for each condition. Statistical analysis was performed using one-way ANOVA. Error bars indicate S.E.M. *p* values between Lphn3-WT and control (Ctrl) or S810L data are indicated by *: **** *p* ≤ 0.0001.

Single-cell migration in cancer is underlined by a change in the expression profile of adhesion molecules in a process that can lead to endothelial-mesenchymal transition, in which cell-cell contacts are lost, thus allowing spreading to occur throughout the organism for the establishment of secondary tumors or metastases in other tissues from a primary cancerous tumor [47]. It is noteworthy that all Lphn3 cancer-related mutants described here were identified in primary tumors. Given the inhibitory effects of Lphn3 on collective cell migration, we sought to characterize Lphn3 cell-autonomous effects by monitoring single-cell motility parameters using time-lapse fluorescence microscopy imaging of notoriously slow migrating HEK293 cells [48]. In an attempt to first discriminate between gain-of-function versus loss-of-function motility phenotypes originating from Lphn3 expression, we tracked the movement of cells harboring the receptor in the absence or presence of its agonist ligand Flrt3. The stable displacement velocity displayed by Lphn3-expressing cells exhibited a sudden burst immediately after the addition of soluble Flrt3 into the cell medium (Figure 8a–d). However, this increase in velocity was transient as cells reached pre-incubation displacement velocity levels after 50 min (Figure 8e,f, Supplementary Video S1). Thus, with this information in hand it can be concluded that increased receptor activation corresponds to increased cell velocity. We then sought to characterize the effect of constitutive receptor activity on the movement of cells. When the trajectories of Lphn3-expressing cells were tracked and compared to control mVenus-expressing cells, they consistently exhibited an increase in distance traveled and consequently possessed higher velocity as well as displayed a change in distancing from the point of origin (Euclidean distance) (Figure 8g–i,k–m). Control mVenus-expressing cells adopted a mesenchymal-like movement characterized by the presence of lamellipodia in contrast to cells expressing Lphn3, for which cell rounding was evident throughout the period analyzed and bleb structures gave sudden velocity bursts (Supplementary Videos S2 and S3). Based on its inherent ability to induce mostly control-like actin-dependent cell protrusions, such as lamellipodia and a deficiency in G13 protein signaling, the S810L mutant was further analyzed. We observed displacement parameters that departed from the ones describing Lphn3-WT expressing-cells since expression of this mutant receptor led to lower displacement values reflected by control-like traveled distances, velocities and Euclidean distances (Figure 8j,k–m, Supplementary Video S4). Therefore, the cell-autonomous effect induced by the mutant receptor’s expression consisted in a loss-of-function phenotype, which was in line with an increase in cell-matrix attachment structures and loss of G protein signaling efficiency both contrasting with Lphn3-WT functions. These results provide evidence that the integrity of Lphn3 GAIN domain, unless disrupted by the presence of select somatic mutations, allows the receptor to act as a modulator of cell motility patterns by regulating cyto-mechanical properties.

## 4. Hijacking Lphn3 Functions to Enhance Pro-Tumorigenic and Pro-Metastatic Cellular Attributes

Throughout the present study, it is noteworthy that the sole expression of Lphn3 in HEK293 cells induces cellular and molecular events that are reminiscent of pro-tumorigenic and pro-metastatic states. First, we report that when lamellipodia-rich HEK293 cells express Lphn3, they reduce their lamellipodia content and adopt morphological characteristics, which are reminiscent of an amoeboid phenotype known to essentially consist of a rounded cell morphology with reduced cell-matrix contacts, and which was described as being an important cell transition state supporting cancer cells migration when abandoning their initial mesenchymal lamellipodia-rich morphology to invade other tissues [46,49,50]. Second, Lphn3-expressing cells acquire a more rapid motility pattern, a feature that has been shown to support the amoeboid migration in cancer and is suspected to confer a higher level of invasion aggressiveness during the metastatic process [51]. Third, distribution of the cancer cell-transition marker vimentin was robustly reorganized while also inducing nuclear deformations in the presence of Lphn3 expression [52,53]. Fourth, the modulation of G13 protein activity, which we reported to be induced by Lphn3 intrinsic signaling, was described as constituting a central part for the progression of various cancers by instructing cell proliferation, invasion and metastasis [54,55,56]. Finally, Lphn3 expression was able to influence collective cell migration possibly by exerting an impairment in cytokine and growth factors cell response and/or secretion, suggesting a modulation of the cells’ microenvironment, in particular inflammation mechanisms known to be contributing to cancer progression [57]. In support of such a role for Lphn3, its overexpression in specific cancerous cells has been observed to correlate with altered cellular properties facilitating cancer progression [27]. The observation that cancer-related GAIN domain mutants studied here share some of Lphn3 attributes further supports the idea that fundamental receptor properties can be hijacked while additional functions are conferred by the specific mutations in order to change the course of cancer progression. In particular, the alteration of G13 signaling seen in all but one cancer-related Lphn3 variants indicates that non-synonymous single amino acid substitutions targeting the GAIN domain can singlehandedly modify its canonical signaling properties, potentially establishing a successful strategy to reprogram the adhesion-sensing capabilities of cancer cells. Moreover, the loss of intercellular contacts through heterophilic interactions between Lphn3 GAIN variants and Flrt3, instead of Teneurin2, highlights the presence of a ligand-specific component potentially inducing a preferential weakening of olfactomedin-based adhesion complexes, rather than lectin-mediated contacts. Notably, lung tissues are reported to display almost undetectable levels of Teneurin protein expression while maintaining a high Flrt3 expression, thus making Lphn3-mediated cell adhesions particularly vulnerable to destabilizing-mutations affecting this ligand’s function in this tissue [58]. The same holds true for the prostate and intestine, where the A760G variant was reported in cancer samples and where differential expression between Teneurin2 and Flrt3 could introduce a tissue-specific component to the change of cell adhesion profile [59,60]. While the factors instructing mesenchymal-to-amoeboid transition are unknown, we propose that Lphn3′s function when over-expressed can lower the plasticity threshold and tip the balance to facilitate this type of cellular transition. Meanwhile, somatic mutations in the GAIN domain can fine-tune the receptor’s impact on cell-cell or cell-matrix adhesion, allowing for a group of cells to be isolated from the rest through weakening intercellular contacts with intact tissue, thus potentially leading to tumor formation or metastasis. However, given that these receptor mutants were studied in a different cellular context (HEK293 cells) than their originating tissues, additional studies are needed to dissect the steps that are triggered by Lphn3 GAIN domain variants in the development of carcinogenesis.

## 5. Conclusions

Our findings highlight functional contributions of the aGPCR signature GAIN domain into determining cellular morpho-mechanical properties by proxy analysis of cancer-related Lphn3 variants bearing a non-synonymous single amino acid substitution. The deficiency in G protein signaling and cell motility reported here for these GAIN domain variants in Lphn3, and the report of cancer-related mutations in other aGPCRs targeting the same region, allude to a similar yet distinct function of this autoproteolysis-prone structural domain. Through our analysis, we propose that G protein-mediated constitutive activity of aGPCRs might rely on the conformational integrity of the GAIN domain, which may act as a structural hub for receptor signaling. Moreover, the GAIN domain can exert an orthosteric modulation of the function of seemingly distant adhesion domains despite it not being directly involved or being necessary for the initial formation of heterophilic intercellular protein-protein interactions. Therefore, tumorigenesis events involving GAIN domain mutations are likely to modify the adhesion and migratory profiles of cancer cells, meanwhile hijacking aGPCRs’ functions to lower the threshold necessary for oncogenic transition states.

## Data Availability

Plasmids can be made available upon request.

## Supplementary Materials

The following supporting information can be downloaded at: https://www.mdpi.com/article/10.3390/cells11121913/s1, Figure S1: Flrt3 binding is restricted to cells expressing Lphn3 or its variants; Figure S2: Extended data for immunoblot images; Figure S3: Vimentin rearrangement in Lphn3-expressing cells coincides with nuclear deformations; Figure S4: Flrt3 does not act as a migration-inducing agent in wound healing assays but expression of Lphn3 or cancer-associated GAIN variants cell-autonomously reduces the migration potential of HEK293 cells; Video S1: Time-lapse fluorescence confocal microscopy imaging of HEK293 cells expressing mVenus-tagged Lphn3-WT stimulated with Flrt3; Video S2: Time-lapse fluorescence confocal microscopy imaging of HEK293 cells expressing mVenus; Video S3: Time-lapse confocal microscopy fluorescence imaging of HEK293 cells expressing mVenus-tagged Lphn3-WT; Video S4: Time-lapse confocal microscopy fluorescence imaging of HEK293 cells expressing mVenus-tagged S810L mutant receptor. References [29,30,31,61,62,63,64,65] are cited in Supplementary Materials.
