# Supplementary material for: Thwarting of Lphn3 Functions in Cell Motility and Signaling by Cancer-Related GAIN Domain Somatic Mutations
Source: Cells. 2022 Jun 13;11(12):1913. doi: 10.3390/cells11121913 (PMC9221416; doi:10.3390/cells11121913)
Supplement: Supplementary file 1 [file cells-11-01913-s001.zip › Cell line choice justification.pdf]

Cell line choice justification:

The HEK293 cell line has been extensively used to study the cellular and molecular processes induced by GPCRs due to many desirable characteristics like possessing a high growth rate, a complete machinery for incorporating post-translational modifications into GPCRs and are a powerful vehicle for the expression of recombinant proteins due to their high transfection efficiency [61]. In our laboratory we have characterized at different levels the functions of Lphns using HEK293 cells as a heterologous model given that this cell line does not express functional latrophilins or any of their known ligands which makes them suitable to evaluate the effects induced by the expression of this family of aGPCRs [29–31]. Using HEK293 cells, we have characterized the effects of Lphn3 on actin cytoskeleton remodeling, resulting in our group reporting a distinct function of this receptor on the different actin-dependent extensions such as filopodia, lamellipodia and blebs as well as their coupling to G proteins. Interestingly HEK293 express epithelial and mesenchymal protein markers that are regulated during the epithelial-mesenchymal transition process where the involvement of the actin cytoskeleton is also crucial for carcinogenesis [65,66]. The HEK293 cell line has also been used as a model to evaluate pro-oncogenic characteristics induced by genes related to carcinogenesis [67,68]. In view of existing antecedents, the use of HEK293 cells solidifies our understanding of Lphn3 cancer-associated mutations in cellular events such as adhesion, signaling and migration, all of which actively involves the actin cytoskeleton and which undergo a strict regulation in oncogenesis. Therefore, we consider that the use of this cell line is justified and can be viewed as a tried-and-true system for the study of GPCRs.

61. Thomas, P.; Smart, T.G. Hek293 cell line: A vehicle for the expression of recombinant proteins. *Journal of pharmacological and toxicological methods* **2005**, *51*, 187–200.
29. Cruz-Ortega, J.S.; Boucard, A.A. Actin cytoskeleton remodeling defines a distinct cellular function for adhesion g protein-coupled receptors adgrl/latrophilins 1, 2 and 3. *Biology open* **2019**, *8,* bio039826.
31. Ovando-Zambrano, J.C.; Arias-Montano, J.A.; Boucard, A.A. Alternative splicing event modifying adgrl1/latrophilin-1 cytoplasmic tail promotes both opposing and dual camp signaling pathways. *Ann N Y Acad Sci* **2019**, *1456*, 168–185.
30. Moreno-Salinas, A.L.; Holleran, B.J.; Ojeda-Muñiz, E.Y.; Correoso-Braña, K.G.; Ribalta-Mena, S.; Ovando-Zambrano, J.C.; Leduc, R.; Boucard, A.A. Convergent selective signaling impairment exposes the pathogenicity of latrophilin-3 missense variants linked to inheritable adhd susceptibility. *Mol Psychiatry* **2022,** *27*, 2425–2438.
65. Inada, M.; Izawa, G.; Kobayashi, W.; Ozawa, M. 293 cells express both epithelial as well as mesenchymal cell adhesion molecules. *International journal of molecular medicine* **2016**, *37*, 1521–1527.
66. Biskou, O.; Casanova, V.; Hooper, K.M.; Kemp, S.; Wright, G.P.; Satsangi, J.; Barlow, P.G.; Stevens, C. The type iii intermediate filament vimentin regulates organelle distribution and modulates autophagy. *PLoS One* **2019**, *14*, e0209665.
67. Stepanenko, A.A.; Dmitrenko, V.V. Hek293 in cell biology and cancer research: Phenotype, karyotype, tumorigenicity, and stress-induced genome-phenotype evolution. *Gene* **2015**, *569*, 182–190.
68. Jin, J.; Woodgett, J.R. Chronic activation of protein kinase bbeta/akt2 leads to multinucleation and cell fusion in human epithelial kidney cells: Events associated with tumorigenesis. *Oncogene* **2005**, *24*, 5459–5470.
