# Supplementary material for: Thwarting of Lphn3 Functions in Cell Motility and Signaling by Cancer-Related GAIN Domain Somatic Mutations
Source: Cells. 2022 Jun 13;11(12):1913. doi: 10.3390/cells11121913 (PMC9221416; doi:10.3390/cells11121913)
Supplement: Supplementary file 1 [file cells-11-01913-s001.zip › Supplementary figure and video legends.pdf]

Supplementary Figure Legends

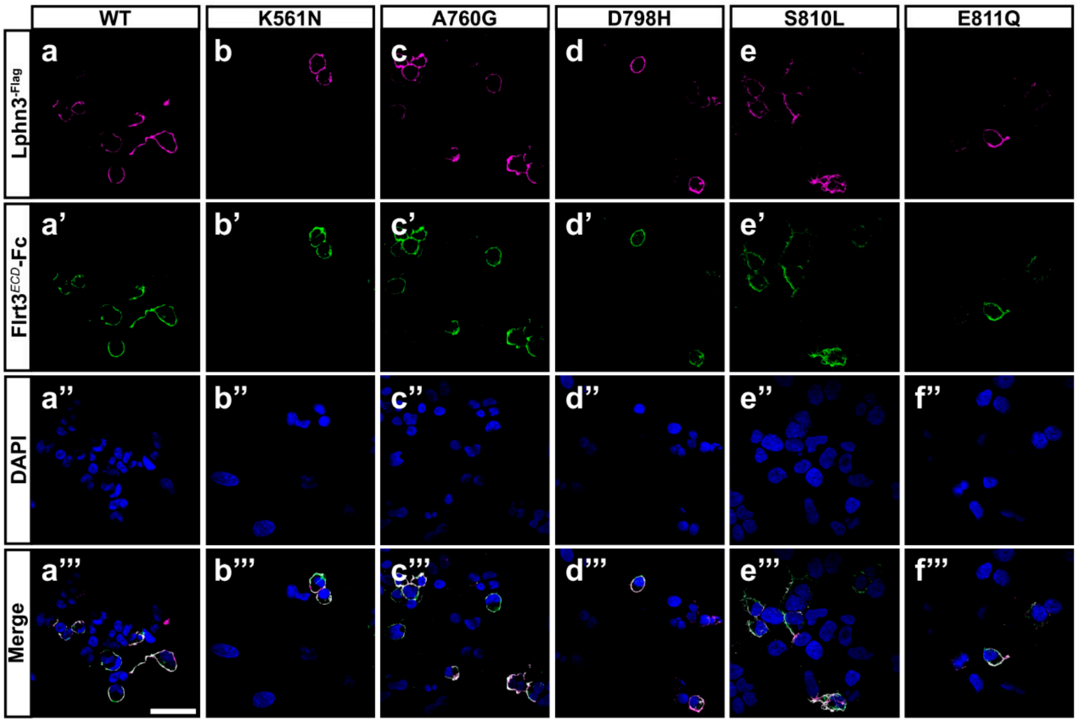

Supplementary Figure S1. Flrt3 binding is restricted to cells expressing Lphn3 or its variants. (**a**-**f**) Representative images obtained by fluorescence confocal microscopy of HEK293 cells expressing Lphn3 or its cancer-related variants (magenta) that were incubated (**a′**-**f′**) in the presence of Flrt3^{ECD}-Fc consisting of the extracellular region of Flrt3 fused to the constant fraction of IgG (green). (**a′′**-**f′′**) Nuclei stained with DAPI. (**a′′′**-**f′′′**) Merge images from corresponding series. Scale bar 50 μm.

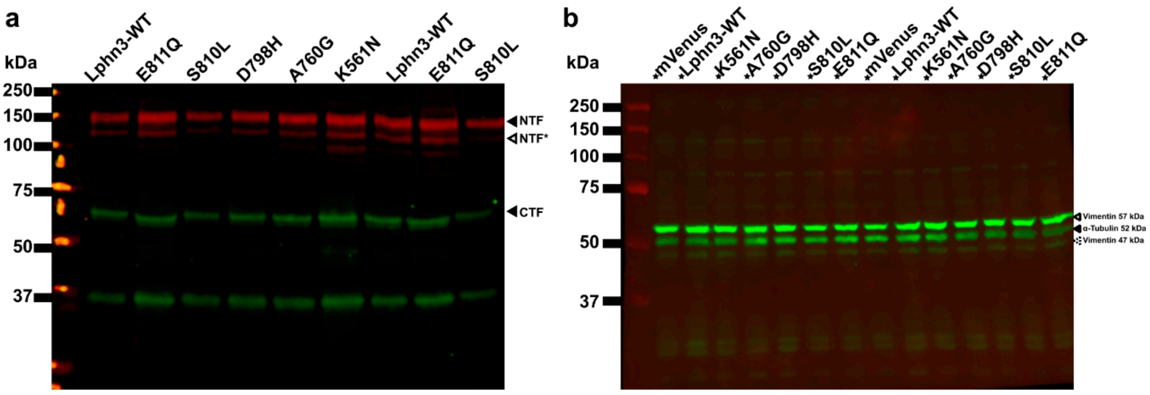

Supplementary Figure S2. Extended data for immunoblot images. (**a**) Representative full-sized immunoblot from data shown in figure 3a analyzing total lysates of HEK293 cells transfected with Lphn3 and the different cancer-associated variants. The amino-terminal portion (NTF) was immunodetected using anti-Flag tag antibody and the carboxyl-terminal end (CTF) with the anti-hemagglutinin tag antibody. (**b**) Representative full-sized immunoblot from data shown in figure 6n analyzing total lysates of HEK293 cells transfected with Lphn3 and the different cancer-associated variants. Vimentin was detected using an antibody recognizing its carboxyl-terminal portion. mVenus-tagged receptor constructs are indicated by a star (★).

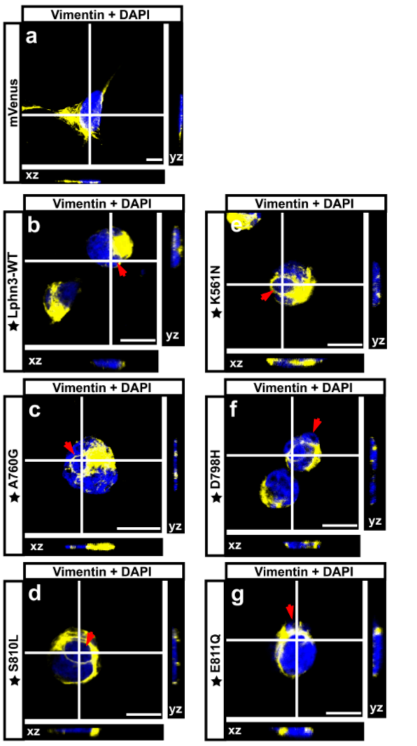

Supplementary Figure S3. Vimentin rearrangement in Lphn3-expressing cells coincides with nuclear deformations. (**a**-**g**) Representative Z-dimension images displaying the vimentin cytoskeleton (yellow) and nuclei (blue) of HEK293 cells expressing Lphn3 or its variants fused to mVenus. Vimentin was immunodetected using an antibody that recognizes its carboxyl-terminal region and DAPI for nuclei. Control cells were transfected with mVenus plasmid alone. White lines in the central image indicate the orthogonal planes of XZ (bottom panel) and YZ (right panel). Arrows represent nuclear lobulations that coincide with the presence of surrounding vimentin-like rings. It is noteworthy that control cell nuclei were not surrounded by vimentin structures. At least 30 cells were analyzed for each condition in three independent experiments. Scale bar: 10 μm. mVenus-fused receptor constructs are indicated by a star (★).

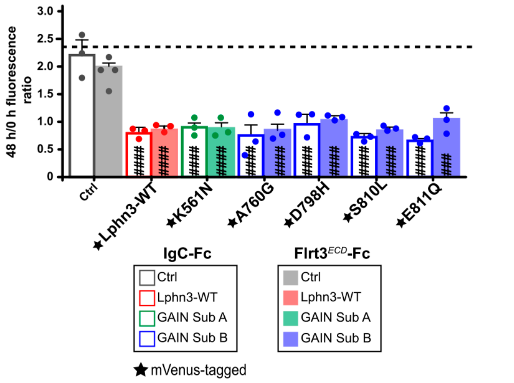

Supplementary Figure S4. Flrt3 does not act as a migration-inducing agent in wound healing assays but expression of Lphn3 or cancer-associated GAIN variants cell-autonomously reduces the migration potential of HEK293 cells. The area occupied by cells expressing mVenus-tagged receptors or mVenus alone was quantified before (0 h) and 48 h after the addition of Ig-Fc or $Flrt3^{ECD}$-Fc (10 nM) and expressed as a ratio of fluorescence area at 48 h over fluorescence area at 0 h. Note that while mVenus-expressing cells gave ratios of 2, suggesting cell migration into the scratch area, Lphn3-WT and receptor variants-expressing cells gave ratios of 1 suggesting the absence of migration independently of Flrt3 presence. Area quantifications were performed with ImageJ software. The dotted line represents values obtained for the positive control. Subdomain A (GAIN Sub A) and B (GAIN Sub B) of GAIN domain. mVenus-tagged receptor constructs are indicated by a star (★). Data are represented as the mean values of at least three independent experiments (n=3). Error bars indicate S.E.M. *P* values between mVenus-fused Lphn3 receptors and control mVenus data are indicated by #: $^{\#\#\#\#}$ P≤0.0001, $^{\#\#\#}$ P≤0.001, $^{\#\#}$ P≤0.01, $^{\#}$ P≤0.05.

Supplementary Video Captions

Supplementary Video S1. Time-lapse fluorescence confocal microscopy imaging of HEK293 cells expressing mVenus-tagged Lphn3-WT stimulated with Flrt3. Images were acquired 43 h after transfection, capturing one image every 5 min for a one-hour time-period, at which point FLRT3^{ECD}-Fc was added and image acquisition resumed for an additional hour. Video editing was achieved by collating 1 image per second into a continuous series, each frame corresponding to an image acquired at 5 min interval. Cell trajectories were obtained with the MTrackJ plugin of Fiji-ImageJ.

Supplementary Video S2. Time-lapse fluorescence confocal microscopy imaging of HEK293 cells expressing mVenus. Images were acquired 43 h after transfection, capturing one image every 10 min for a three-hour time-period. Video editing was achieved by collating 1 image per second into a continuous series, each image corresponding to a 10 min interval frame. Cell trajectories were obtained with the MTrackJ plugin of Fiji-ImageJ

Supplementary Video S3. Time-lapse confocal microscopy fluorescence imaging of HEK293 cells expressing mVenus-fused Lphn3-WT. Images were acquired 43 h after transfection, capturing one image every 10 min for a three-hour time-period. Video editing was achieved by collating 1 image per second into a continuous series, each image corresponding to a 10 min interval frame. Cell trajectories were obtained with the MTrackJ plugin of Fiji-ImageJ

Supplementary Video S4. Time-lapse confocal microscopy fluorescence imaging of HEK293 cells expressing mVenus-tagged S810L mutant receptors. Images were acquired 43 h after transfection, capturing one image every 10 min for a three-hour time-period. Video editing was achieved by collating 1 image per second into a

continuous series, each image corresponding to a 10 min interval frame. Cell trajectories were obtained with the MTrackJ plugin of Fiji-ImageJ
